# 6-Bromoindirubin-3′-Oxime Regulates Colony Formation, Apoptosis, and Odonto/Osteogenic Differentiation in Human Dental Pulp Stem Cells

**DOI:** 10.3390/ijms23158676

**Published:** 2022-08-04

**Authors:** Chatvadee Kornsuthisopon, Sunisa Rochanavibhata, Nunthawan Nowwarote, Kevin A. Tompkins, Waleerat Sukarawan, Thanaphum Osathanon

**Affiliations:** 1 Dental Stem Cell Biology Research Unit, Faculty of Dentistry, Chulalongkorn University, Bangkok 10330, Thailand; 2 Department of Oral and Maxillofacial Surgery, Faculty of Dentistry, Chulalongkorn University, Bangkok 10330, Thailand; 3 Department of Oral Biology, Faculty of Dentistry, Universite Paris Cite, 75006 Paris, France; 4 Centre de Recherche des Cordeliers, INSERM UMRS 1138, Molecular Oral Pathophysiology, Universite Paris Cite, Sorbonne Universite, 75006 Paris, France; 5 Office of Research Affairs, Faculty of Dentistry, Chulalongkorn University, Bangkok 10330, Thailand; 6 Department of Pediatric Dentistry, Faculty of Dentistry, Chulalongkorn University, Bangkok 10330, Thailand; 7 Department of Anatomy, Faculty of Dentistry, Chulalongkorn University, Bangkok 10330, Thailand

**Keywords:** BIO, dental pulp stem cells, odonto/osteogenic differentiation, Wnt/β-catenin

## Abstract

6-bromoindirubin-3′-oxime (BIO) is a candidate small molecule that effectively modulates Wnt signalling owing to its stable property. The present study investigated the influence of BIO on the odonto/osteogenic differentiation of human dental pulp stem cells (hDPSCs). hDPSCs were treated with 200, 400, or 800 nM BIO, and the effects on hDPSC responses and osteogenic differentiation were assessed. BIO-mediated Wnt activation was confirmed by β-catenin nuclear translocation detected by immunofluorescence staining. BIO attenuated colony formation and cell migration determined by in vitro wound-healing assay. BIO increased early apoptotic cell population evaluated using flow cytometry. For osteogenic induction, BIO promoted alkaline phosphatase (ALP) activity and mineralisation in a dose-dependent manner. *ALP*, *RUNX2*, *OCN*, *OSX*, *ANKH*, *DMP1*, and *DSPP* mRNA expression were significantly upregulated. The OPG/RANKL expression ratio was also increased. Further, BIO attenuated adipogenic differentiation as demonstrated by decreased lipid accumulation and adipogenic-related gene expression. Bioinformatic analysis of RNA sequencing data from the BIO-treated hDPSCs revealed that BIO modulated pathways related to autophagy and actin cytoskeleton regulation. These findings demonstrated that BIO treatment promoted hDPSC osteogenic differentiation. Therefore, this small molecule is a strong candidate as a bioactive molecule to enhance dentin repair.

## 1. Introduction

Clinical restorative procedures aim to preserve dental pulp vitality and, in some cases, promote dentin bridge formation [1]. Direct pulp capping using a pulp-capping material is commonly performed to generate a reparative dentin [2]. The failure of a material to degrade and an inflammatory reaction are the major concerns for materials currently used clinically [3]. These can lead to incomplete dentin restoration and cell necrosis [4]. Thus, unsatisfactory outcomes, including treatment failure, occur. 

In response to dental pulp injury, mesenchymal stem cells (MSCs) in dental pulp tissues proliferate and migrate to the injured area [5]. Further, these cells differentiate into odontoblast-like cells that subsequently secrete dentin matrix to support pulp healing and dentin regeneration [6,7]. The concept of biologically active agents for preserving dental pulp vitality was introduced [8]. Previous studies have demonstrated that various bioactive molecules were employed as potential bioactive agents for the tertiary dentin stimulation [9,10,11]. However, these soluble growth factors have been shown to induce the formation of excessive osteodentin, pulp chamber obliteration, and pulp stone formation. 

The Wnt signalling pathway is an essential signalling cascade that regulates developmental events during embryogenesis and tissue homeostasis in adults [12]. This pathway modulates many cellular events in dental pulp cells, including the stem cell differentiation [13]. Wnt signalling influences the epigenetic control of human dental pulp stem cells (hDPSCs) and modulates stemness maintenance, self-renewal, and differentiation of these stem cells [14]. Moreover, the metabolism of hDPSCs was reprogrammed using Wnt agonists, 6-bromoindirubin-3′-oxime (BIO) and WNT-3A, resulting in enhancing dental pulp stem cell stemness and pluripotency [15]. Ferutinin, the compound that activated the Wnt signalling pathway, promoted the osteogenic differentiation capacity of hDPSCs [16]. This evidence suggested the importance of Wnt signalling in controlling cellular events and epigenetics of hDPSCs.

Previous studies have indicated that Wnt ligands promote pulp healing [17,18,19] and odonto/osteogenic differentiation in dental-related MSCs [20,21]. Direct pulp capping in animal teeth activates canonical Wnt signalling, defined by nuclear translocation of β-catenin in odontoblast-like and dental pulp cells beneath the injured site [22,23]. Similarly, Wnt responsive cells defined by expressing Axin2 proliferated and differentiated into odontoblast-like cells following pulp exposure. Inhibition of Wnt signalling in Axin2-expressing cells compromised the process of reparative dentin formation. These results indicated that these Wnt-responsive cells secreted Wnt ligands to induce dentin repair by the autocrine Wnt/β-catenin signalling [18]. This evidence implied the involvement of canonical Wnt signalling in odontoblast differentiation during reparative dentin formation. However, the use of recombinant Wnt protein in clinical treatment materials is limited due to the unstable and highly hydrophobic nature of Wnt proteins [24,25]. Given the stable properties of small molecules, modulating Wnt signalling by combining pulp-capping materials with small molecules is considered an intriguing treatment modality to promote dentin bridge formation. BIO is a small molecule that targets the glycogen synthase kinase-3 (GSK-3) [26]. A previous report demonstrated that BIO enhanced alkaline phosphatase (ALP) activity and osteogenesis-related gene expression in osteoprogenitor cells [27]. Similarly, BIO accelerated the in vitro chondrogenesis of mouse bone marrow mesenchymal stem cells (BMSCs), demonstrated by the upregulation of cartilage-specific gene expression [28]. Additionally, BIO promoted reparative dentin formation at the injured site compared with the conventional collagen sponges [29]. However, the effect of BIO on the odonto/osteogenic differentiation in hDPSCs in vitro remains unresolved. Thus, the present study aimed to investigate BIO′s influence on hDPSC odonto/osteogenic differentiation in vitro and further evaluate the signalling mechanism using a high-throughput RNA sequencing analysis.

## 2. Results

### 2.1. Cell Characterisation

**Figure 1:**
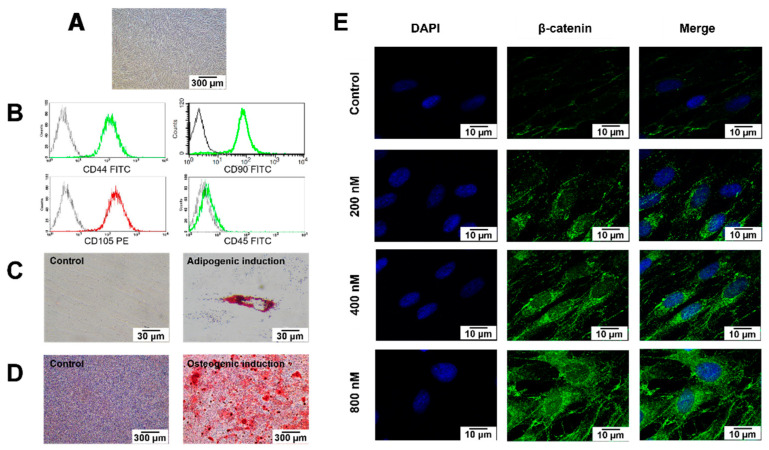
Characterisation of the cells isolated from dental pulp tissues. (**A**) Morphological observation of human dental pulp stem cells (hDPSCs) using phase-contrast microscopy. Scale bars: 300 µm. (**B**) Evaluation of stem cell surface markers using flow cytometry. (**C**) Multi-lineage differentiation potential toward adipogenic (**D**) and osteogenic lineage. Scale bars: 30 and 300 µm. Intracellular lipid accumulation was stained by Oil Red O staining. Calcium accumulation was stained using Alizarin Red S (ARS) staining. (**E**) Representative images of immunofluorescence staining of β-catenin (stained in green) in hDPSCs; nuclei were counterstained with DAPI (shown in blue). White arrows indicate the increased nuclear translocation of β-catenin. Scale bars: 10 µm.

The isolated cells (passage 4) had a spindle-shaped, fibroblast-like morphology (Figure 1A). Flow cytometry analysis revealed the expression of MSC-related surface markers (CD44, CD90, and CD105) and the absence of the hematopoietic cell marker CD45 (Figure 1B). After adipogenic induction, intracellular lipid accumulation markedly increased compared with cells cultured in a growth medium (Figure 1C). Similarly, hDPSCs maintained in an osteogenic medium exhibited increased mineral deposition compared with the undifferentiated control cells (Figure 1D).

### 2.2. BIO Treatment Activated Wnt Signalling in hDPSCs

The subcellular localization of β-catenin was examined using immunofluorescence staining to confirm that BIO activated the Wnt pathway. The cells were cultured in a growth medium composed of Dulbecco’s Modified Eagle Medium that contained 10% fetal bovine serum, 2 mM L-glutamine, 100 unit/mL penicillin, 100 μg/mL streptomycin, and 250 ng/mL amphotericin B. In total, 200 nM, 400 nM, or 800 nM BIO was added into the growth medium and the cells were maintained in the culture medium for 24 h. The cells cultured in a growth medium were used as the control. BIO-treated hDPSCs demonstrated increased cytoplasmic β-catenin accumulation and nuclear translocation compared with those cultured in the normal growth medium (Figure 1E).

### 2.3. BIO Attenuated hDPSC Colony-Forming Unit Ability and Cell Migration by Inducing Early Apoptosis

**Figure 2:**
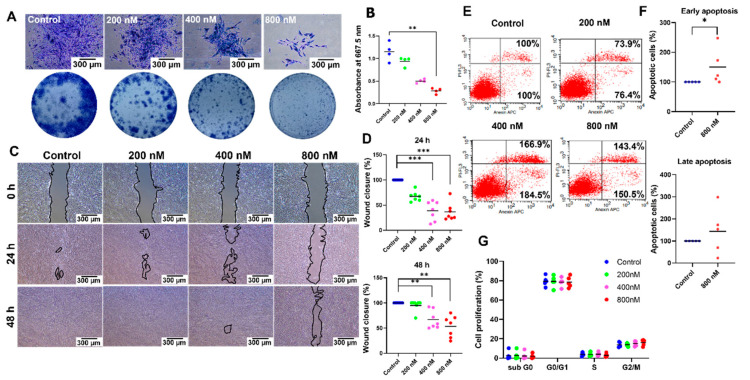
6-bromoindirubin-3′-oxime (BIO) attenuates hDPSC colony-forming unit ability and cell migration by inducing early apoptosis. (**A**) Colony-forming unit assay. Scale bars: 300 µm. (**B**) The staining was solubilised, and the absorbance was determined. (**C**) Representative images of the in vitro scratch assay at 0, 24, and 48 h. Scale bars: 300 µm. (**D**) Quantification results of the percentage migration. (**E**,**F**) Flow cytometry analysis of the apoptotic cells. The left upper and lower panels show dead cells and live cells, respectively. Right upper and lower panels indicate late apoptotic cells and early apoptotic cells, respectively. (**G**) Cell cycle analysis was performed using flow cytometry. Bars indicate a significant difference between groups (* *p* < 0.05. ** *p* < 0.01. *** *p* < 0.001).

BIO-treated hDPSCs exhibited reduced colony-forming size and density compared with the control hDPSCs in a dose-dependent manner (Figure 2A). A significant difference was detected when the cells were exposed to 800 nM BIO, as illustrated in the staining quantification (*p* = 0.0050) (Figure 2B). Moreover, significantly delayed wound closure was observed in 400 nM and 800 nM BIO treatment groups compared with the control group at 24 (*p* = 0.0006 each) and 48 h (*p* = 0.0083, *p* = 0.0011; respectively) (Figure 2C,D). To investigate the mechanism responsible for these results, the number of apoptotic cells was detected using flow cytometry. Increased early and late apoptotic cells were found in the cells treated with BIO compared with the non-treated cells. However, a significant difference was detected only in the early apoptotic phase in hDPSCs treated with 800 nM BIO (*p* = 0.0476) (Figure 2E,F). Cell cycle analysis was also performed using flow cytometry. The results demonstrated that BIO treatment exhibited a non-significant tendency to cell cycle regulation (Figure 2G).

### 2.4. BIO Promoted Odonto/Osteogenic Differentiation but Attenuated Adipogenic Differentiation of hDPSCs

**Figure 3:**
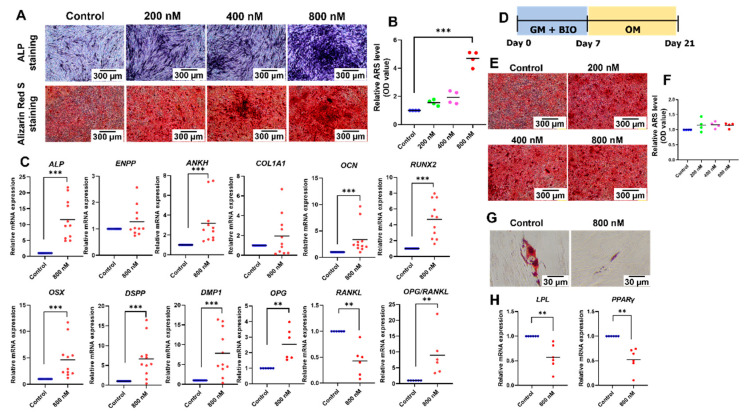
BIO promotes odonto/osteogenic differentiation but attenuates adipogenic differentiation of hDPSCs. (**A**) Osteogenic differentiation was determined by alkaline phosphatase (ALP) staining and mineral deposition. Scale bars: 300 µm. (**B**) Quantifying ARS staining. (**C**) The mRNA levels of osteogenic-related marker genes. (**D**) Timeline representing the pretreatment effect experiment. (**E**) Osteogenic differentiation was determined by mineral deposition. Scale bars: 300 µm. (**F**) Quantification of ARS staining. (**G**) Adipogenic differentiation was evaluated by intracellular lipid deposition. Scale bars: 30 µm. (**H**) The mRNA levels of adipogenic-related marker genes. Bars indicate a significant difference between groups (** *p* < 0.01. *** *p* < 0.001).

To evaluate the influence of BIO on osteogenic differentiation, the cells were maintained in an osteogenic medium supplemented with 200 nM, 400 nM, or 800 nM BIO. The cells cultured in an osteogenic medium were used as the control. After osteogenic differentiation, BIO treatment enhanced ALP staining and mineral deposition in a dose-dependent manner (Figure 3A). Quantifying the Alizarin Red S staining revealed that 800 nM BIO significantly upregulated mineral deposits (Figure 3B). Osteogenic marker gene expression was evaluated on day 7. Treatment with 800 nM BIO significantly induced *ALP*, *ANKH*, *OCN*, *RUNX2*, *OSX*, *DSPP*, *DMP1*, and *OPG* mRNA expression (*p*  =  0.0001, *p* = 0.0001, *p* = 0.0002, *p* = 0.0001, *p* = 0.0001, *p* = 0.0002, *p* = 0.0002, *p* = 0.0022; respectively) (Figure 3C). The expression level of *RANKL* mRNA was significantly decreased by 800 nM BIO (*p* = 0.0022) (Figure 3C). Hence, the mRNA expression ratio of *OPG* and *RANKL* was upregulated considerably (*p* = 0.0022) (Figure 3C). To determine whether BIO pretreatment affected osteogenic differentiation, the cells were maintained in a growth medium supplemented with 200 nM, 400 nM, or 800 nM BIO for 7 d and then cultured in an osteogenic medium without BIO supplementation for 14 d (Figure 3D). No difference in mineral deposition was observed between these culture conditions (Figure 3E,F). 

For adipogenic differentiation, decreased intracellular lipid accumulation was observed in the BIO-treated group (Figure 3G). The mRNA expression of adipogenic marker genes, *LPL* and *PPARγ*, were evaluated on day 8. BIO treatment at 800 nM significantly downregulated *LPL* and *PPARγ* mRNA expression (*p*  = 0.0022 each) (Figure 3H).

### 2.5. Gene Expression Profile of the BIO-Treated hDPSCs

**Figure 4:**
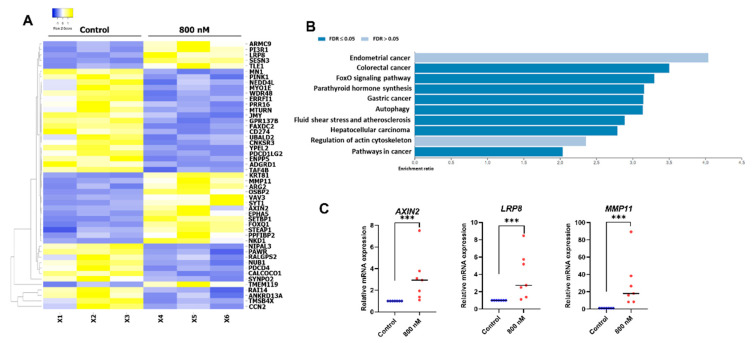
BIO-treated hDPSC gene expression profile. (**A**) RNA sequencing analysis was performed. Heatmap showed the top 50 significant differentially regulated genes. (**B**) KEGG pathway database enrichment analysis for the differentially expressed genes was performed by WebGestalt. (**C**) The differential gene expression of *AXIN2*, *LRP8*, and *MMP11* was confirmed using a real-time polymerase chain reaction. Bars indicate a significant difference between groups (*** *p* < 0.001).

hDPSCs were treated with 800 nM BIO and maintained in a growth medium for 24 h. The cells cultured in a normal growth medium were used as a control. Total cellular RNA was isolated and analysed for global differential gene expression compared with the control using RNA sequencing. The expression pattern of related genes is illustrated as a heatmap (Figure 4A). Based on the KEGG pathway database enrichment analysis, the differential expressed genes were classified into several pathways, including pathways related to autophagy and regulation of the actin cytoskeleton (Figure 4B). *AXIN2*, *LRP8*, and *MMP11* mRNA levels were selected to validate the RNA sequencing results using real-time quantitative polymerase chain reactions. Significant upregulation of those genes was found after 800 nM BIO treatment (*p* = 0.0006 each) (Figure 4C).

## 3. Discussion

In the present study, we have shown that BIO inhibited colony formation and cell migration. Early cell apoptosis was increased after BIO stimulation. BIO treatment induced the differentiation of hDPSCs towards the osteogenic lineage, as demonstrated by increased ALP activity, mineral deposition, and odonto/osteogenic-related gene expression. Downregulation of intracellular lipid accumulation and adipogenic-related mRNA expression indicated decreased adipogenic differentiation. Hence, the present study highlights the influence of BIO on hDPSC cell apoptosis and osteogenic differentiation. 

In the present study, dental pulp cells were isolated from the dental pulp tissue of healthy patients. The MSC characterisation followed the guidelines of the International Society for Cellular Therapy [30]. Their multi-lineage differentiation capacity was exhibited via osteogenic and adipogenic differentiation. As MSCs, dental pulp cells play an essential role in dentin regeneration because they can differentiate into odontoblast-like cells to secrete dentin matrix in response to pulp injury [6,7,31]. Considering their therapeutic potential, dental pulp cells are promising cells for regenerative endodontic therapy and cell therapy in regenerative medicine [32,33].

Multiple signalling pathways function in concert to regulate physiological processes. Wnt signalling is one of the essential signalling cascades that regulate various biological events, including cell proliferation, cell cycle progression, cell fate determination, apoptosis, differentiation, migration, and osteogenic differentiation in multiple tissues [34,35,36]. Canonical Wnt signal transduction involves a β-catenin [37]. BIO is a small molecule Wnt agonist that modulates the canonical Wnt pathway through GSK-3 inhibition, resulting in decreased β-catenin phosphorylation. β-catenin accumulates in the cytosol and translocates into the nucleus, which is the hallmark of the canonical Wnt signaling [26]. In the present study, the effective concentrations of BIO were confirmed by β-catenin nuclear translocation detected by immunohistochemistry staining. The results demonstrated that 200 nM, 400 nM, and 800 nM BIO treatment caused increased cytoplasmic accumulation and nuclear translocation of β-catenin, indicating Wnt signaling pathway activation in hDPSCs. 

In this study, we used the BIO concentration range (200 nM, 400 nM, and 800 nM) that corresponded with previous studies [38]. Colony unit formation is an indicator of cell proliferation. Our study demonstrated that BIO treatment attenuated colony-forming ability in a dose-dependent manner. Concomitantly, compromised migration was observed after treatment with BIO. These results imply impaired cell proliferation or induced cell apoptosis. Abilities of the cells to form colonies and migrate depend on the number of cells that are viable to proliferate and form small colonies [39]. Thus, we speculated that BIO-induced apoptosis led to impaired colony forming unit ability and migration. The effect of BIO on cell proliferation remains unresolved. It was reported that BIO promoted the proliferation of human periodontal ligament stem cells (PDLSCs) [40], rat marrow-derived mesenchymal stem cells [41], and rat cardiomyocytes [42]. In contrast, other studies demonstrated that BIO decreased cell proliferation in stem cells isolated from human exfoliated deciduous teeth [40], hDPSCs [43], ovarian cancer cells [44], a mouse myoblast cell line [45], canine BMSCs [46], and canine melanoma cell lines [47]. Similarly, the effect of Wnt on cell migration remains controversial. Migration and invasion of human melanoma cell lines (GLL-19 cells) were attenuated by SFRP5, which was the extracellular regulator of the Wnt signalling pathway [48]. On the contrary, impaired Wnt/β-catenin signalling pathway mediated by downregulation of Differential embryo-chondrocyte expressed gene 1, a gene that is expressed in most human organs and tissue, attenuated proliferation, migration, and invasion, and induces apoptosis in human ovarian cancer cells [49]. We speculate that the differences in cell response depend on the specific cell type.

A study in a mouse myoblast cell line demonstrated that the cell population in the S phase was reduced following BIO treatment [45]. Wnt-mediated transcription cell factor T-cell factor 1 (TCF1) promoted the expression of INK4, a cyclin-dependent kinase (CDK) inhibitor. Increased expression of INK4 led to inhibition of G1 phase CDKs. Thus, cells remained in the G1 phase, and the number of cells in the S phase decreased [50]. Although the present study demonstrated that BIO treatment led to a slight decrease in the S phase population, the results showed that BIO treatment exhibited a non-significant tendency towards cell cycle regulation. Many components control cell cycle progression, namely the family of cyclin proteins and cyclins and cyclin-dependent kinases. Thus, we hypothesized that there might be several regulator molecules of the cell cycle that might not be affected by BIO treatment, resulting in a non-significant tendency towards cell cycle regulation. BIO treatment increased the percentage of early apoptotic hDPSCs. BIO treatment attenuated apoptosis in various cell types, e.g., colorectal cancer cells [37] and murine embryonic stem cells [51]. However, previous reports in a rat mesangial cell line [52] and human melanoma cells [53] revealed similar results to our study. The Janus kinase-signal transducer and activator of transcription (JAK/STAT) pathway plays a crucial role in the cell survival [54]. The downstream targets of the JAK/STAT pathway include anti-apoptotic protein family members, such as the Bcl-2 family [55]. Inhibiting this pathway promotes the apoptosis [56,57,58]. Mechanistically, BIO functions as a JAK inhibitor that directly targets JAK kinase activity [53]. Thus, BIO might mediate the apoptotic activity through JAK-STAT inhibition. To prove this hypothesis, further investigation is needed, e.g., western blots to detect the level of phosphorylated and total JAK and/or STAT components. In addition, TUNEL and active caspase 3 staining assay should be performed to confirm the effect of BIO-induced apoptosis. However reports showed the participation of apoptotic bodies and mineralization in vitro and skeleton development and bone turnover in vivo. The appropriate time point for apoptotic events is crucial. The present study described that BIO promoted hDPSCs apoptosis and also enhanced osteogenic differentiation and mineralization. However, these experiments were maintained in different medium conditions, the normal growth medium for apoptosis assay and the osteogenic induction medium for differentiation assay. Hence, further investigation regarding the relationship between these two biological events is required.

In the present study, BIO treatment enhanced hDPSC osteogenic differentiation, as seen by increased ALP activity, odonto/osteogenic marker gene expression, and mineralised nodule formation. Similar results were found in other cell types, e.g., human PDLSCs [59], mouse PDLSCs [60], apical papilla stem cells [61], mouse DPs [62], hBMSCs [38], canine BMSCs [46], and mouse embryonic fibroblasts [63]. In contrast, it has been reported that LiCl-mediated Wnt signaling activation decreased the osteogenic differentiation [64,65]. However, topical application of LiCl on the amputated pulp promoted the transdifferentiation of surrounding dental pulp cells toward the odontoblasts, resulting in a dentin regeneration [66]. We speculated that this discrepancy might rely on different cell sources, cell differentiation stages, and culture conditions used in these studies. Previous evidence supported that response to Wnt signalling activation might depend on the specific cell types. BCL9 is a component of nuclear β-catenin that is required for the transcription of endogenous Wnt target genes. This component modulated and diversified the transcriptional responses to Wnt signalling in a cell-type-specific manner, resulting in the different responses to Wnt signalling activation of the specific cell types [67].

Wnt signalling modulates stemness, self-renewal, and differentiation of hDPSCs via epigenetic control. Activation of Wnt/β-catenin signalling by WNT-3A treatment upregulates the pluripotency markers, resulting in genomic DNA demethylation, and increased histone acetylation and histone methylation in hDPSCs [14]. hDPSCs that were pretreated with Wnt agonists, BIO or WNT-3A, exhibited an increase in stemness and differentiation capacity towards osteogenic and adipogenic lineages [14]. Furthermore, BIO and WNT-3A reprogrammed the metabolism of dental pulp stem cells by stimulating mitochondrial metabolism and lipid synthesis, resulting in the accumulation of acetyl-CoA in the nucleus and cytoplasm [15]. Histone acetylation was enhanced by acetyl-CoA, thereby sustaining dental pulp stem cell stemness and pluripotency [68]. Additionally, a compound called Ferutinin activated the Wnt/β-catenin signalling pathway, leading to enhancing the osteogenic differentiation capacity of hDPSCs by inducing expression of osteocalcin and collagen 1A1 in both mRNA and protein levels [16]. These data imply the possibility of enhancing the stemness features of dental pulp stem cells by manipulating the expression of Wnt signalling components, and the importance of Wnt signalling in hDPSC osteogenic differentiation.

Several genes act in concert to control osteogenic differentiation and mineralisation. In our study, we found increased expression of *ALP*, *ANKH*, and *ENPP1* after BIO treatment. These genes control phosphate metabolism and mineralisation. ENPP and ANKH mediate pyrophosphate accumulation. ALP breaks down pyrophosphate into inorganic phosphate, leading to a mineralisation [69,70]. We also observed the upregulation of *RUNX2*, *OSX*, *OCN, DSPP*, *DMP1*, and *OPG* expression following BIO treatment. Similarly, a previous report demonstrated that β-catenin stimulates hDPSC odontoblastic differentiation by mediating the *RUNX2* expression [23]. Runx2 is a master transcription factor that controls the odonto/osteogenic differentiation [71]. This gene regulates the expression of several essential genes for osteoblastic or odontoblastic differentiation, e.g., *DSPP*, *DMP1*, *OSX*, and *OCN* [72,73]. Mechanistically, TCF1, a family of LEF1/TCF transcription factors controlling gene expression downstream of Wnt/β-catenin signalling, occupies the proximal Runx2 promoter. In addition, β-catenin can bind to the Runx2 promoter [63]. Therefore, Wnt activation leads to the upregulation of Runx2 transcription, resulting in the upregulation of osteogenic-related genes and increased mineralisation. 

Several reports demonstrated that Wnt pretreatment led to the enhancement of the osteogenic differentiation [38]. In the present study, hDPSCs were cultured for 7 d in a growth medium containing BIO for 7 d, followed by an osteogenic medium without BIO for 14 d. The results revealed no significant difference in mineralisation with or without BIO pretreatment, suggesting that simultaneous BIO treatment was necessary during osteogenic induction. A similar result was found in hBMSCs [38]. BIO treatment was required during early-stage osteogenesis, and this process can occur simultaneously with terminal differentiation [38]. However, the in-depth investigation would be required to evaluate whether BIO/Wnt signalling regulates osteoblast commitment. In this regard, β-catenin knockout mice using postnasal β-catenin disruption by targeting GSK3 might be an appropriate tool. Evaluation of bone marrow adiposity and bone mass could be performed. Bone marrow-derived cells could be isolated and evaluated for osteogenic differentiation capacity [74]. Alternative to the knockout mice model, RhoA and ROCK are intermediate molecules that determine the mesenchymal stem cell lineage commitment towards osteogenic or adipogenic lineage [75]. Thereby, assessment of the expression of RhoA and ROCK might be an effective procedure to evaluate the effect of BIO on osteoblast commitment.

The RANKL/OPG axis plays a vital role in osteoblastogenesis and osteoclastogenesis, regulating bone formation. RANKL and macrophage colony-stimulating factors regulate the process of osteoclastogenesis. OPG counteracts the action of RANKL, thereby inhibiting the formation of osteoclasts [76]. Previous evidence supported that OPG promoted osteogenesis of human mesenchymal stem cells [77]. β-catenin-knockout mice exhibited significantly downregulated *Opg* mRNA expression, whereas expression of *Rankl* was significantly decreased, leading to a reduction of cortical bone mass. These effects were rescued by the β-catenin activation [78]. Similarly, in osteoporotic postmenopausal patients, serum β-catenin was positively correlated with OPG and negatively correlated with the ratio of RANKL/OPG [79]. Thus, we speculated that BIO-mediated Wnt activation could modulate OPG/RANKL axis. In the present study, we found that BIO treatment upregulated the expression of OPG, whereas RANKL was downregulated. Similarly, a previous study on lithium chloride-coated titanium discs exhibited an increased OPG protein level and a higher OPG/RANKL ratio than the control titanium discs [80]. However, a previous report showed Wnt signalling exerted the regulatory effect only on Opg. Osteocyte-specific β-catenin-deficient mice exhibited downregulation of Opg, while Rankl expression was not affected [81]. Since responses to Wnt signalling occur in a cell-type-specific manner, these result in the different responses to Wnt signalling activation of the specific cell types. Thus, we speculated that the discrepancy in response to Wnt signalling is different due to different cell types.

After adipogenic induction, BIO treatment decreased intracellular lipid accumulation and downregulation of adipogenic-related genes. Similarly, activated Wnt signalling exerted an inhibitory effect on the adipogenesis [82,83,84,85]. The adipogenic transcription factors include CCAAT-enhancer-binding protein α (C/EBPα) and peroxisome proliferator-activated receptor γ (PPARγ) [86]. Wnt signalling attenuates the expression of these two transcription factors, thereby inhibiting the preadipocyte differentiation [87,88]. The underlying molecular mechanism is explained through the β-Catenin-Axin2-GSK3β axis [89]. During Wnt activation, Axin2, a constitutive Wnt target, is upregulated in the cytoplasm. Axin2 binds with GSK3β, preventing nuclear localisation of GSK3β. Thus, GSK3β cannot phosphorylate C/EBPβ and Snail, thereby C/EBPα and PPARγ remain inactivated [89].

The effect of BIO on hDPSC gene expression was assessed using RNA-seq. The results demonstrated that BIO was involved in pathways related to many types of cancer, autophagy, and actin cytoskeleton regulation. In regards to cancer, participation of the Wnt signalling pathway was noted in the development of various kinds of cancer, e.g., lung cancer [90], endometrial cancer [91], and colorectal cancer [92]. Wnt-mediated autophagy was reported [93,94,95]. A previous study of gene expression profile in Wnt3a-treated fibroblast cell lines demonstrated the upregulation of several genes related to fibrogenic pro-adhesive molecules, pro-fibrotic proteins, cell adhesion and migration, vasculature development, and cell proliferation [96]. Correspondingly, canonical Wnt signalling was reported to participate in various cytoskeletal events, e.g., microtubule modulation [97], resulting in regulating cell polarity, cell adhesion, and cell migration [98]. Regarding the developmental process, a frizzled receptor 7-specific Wnt mimetic, Wnt3a, and CHIR regulate the transcriptome of human pluripotent stem cells, including WNT target genes and mesendodermal genes, resulting in endodermal and mesendodermal differentiation [99]. In terms of osteoblastic differentiation, a study on rat calvarial osteoblasts found that Wnt3a regulated the transcriptome in primary osteoblasts. Wnt3a activated the expression of several genes involved in osteoblast proliferation (e.g., *Bmp2*, *Bmp4*, and *Tgfb2*) and the early stage of osteoblastic differentiation (e.g., *Ntf3*, *Enpp1*, and *Col17a1*). Wnt3a also regulated the expression of several transforming growth factor-beta and mitogen-activated protein kinase signalling pathway components, critical regulators of bone development and metabolism [100]. As for odontoblastic differentiation, the polarization of DPSCs is a prerequisite and fundamental step for the cell differentiation [101]. Thus, BIO treatment could mediate cell polarization through actin cytoskeleton regulation, thereby initiating the process of odontoblastic differentiation, which supported our findings that BIO promoted the hDPSC osteogenic differentiation.

## 4. Materials and Methods

### 4.1. Cell Isolation and Culture

Dental pulp tissues were minced, and cell explant was performed. Cells were maintained at 37 °C in a humidified 5% carbon dioxide atmosphere and cultured in a growth medium composed of Dulbecco’s Modified Eagle Medium (DMEM, cat. no. 11960, Gibco, CA, USA) containing 10% fetal bovine serum (FBS, cat. no. 10270, Gibco, CA, USA), 2 mM L-glutamine (GlutaMAX-1, cat. no. 35050, Gibco, CA, USA), 100 unit/mL penicillin, 100 μg/mL streptomycin, and 250 ng/mL amphotericin B (Antibiotic–Antimycotic, cat. no. 15240, Gibco, CA, USA). The culture medium was changed every 48 h. Subsequent assays were performed using the cells from passages 3–7. 

Surface protein expression was determined using flow cytometry. The following antibodies were used at a dilution of 1:50 FITC conjugated anti-human CD44 (Cat. No. 555478, BD Bioscience, San Diego, CA, USA), PE-conjugated anti-human CD105 (Cat. No. 21271054, Immuno Tools, Friesoythe, Germany), APC-conjugated anti-human CD90 (Abcam, San Jose, CA, USA), and FITC-conjugated anti-CD45 (Cat. No. 21810455, Abcam, USA). Mean fluorescence intensity was analysed using a FACS^Calibur^ flow cytometer (BD Bioscience, San Jose, CA, USA).

### 4.2. Differentiation Assays

For odonto/osteoblastic differentiation, cells were seeded at a density of 25,000 cells per well in a 24-well plate and cultured in the osteogenic medium (growth medium supplemented with 50 µg/mL ascorbic acid (cat. no. A-4034, Sigma-Aldrich, CA, USA), 250 nM dexamethasone (cat. no. D8893, Sigma-Aldrich, CA, USA), and 5 mM β-glycerophosphate (cat. no. G9422, Sigma-Aldrich, CA, USA)) for 14 d. ALP staining, mineral deposition, and osteogenic marker gene mRNA expression were investigated using the methods described below.

For adipogenic differentiation, the cells were seeded at a density of 12,500 cells per well in a 24-well plate and maintained in an adipogenic medium (growth medium containing 0.1mg/mL insulin (cat. no. 11070738 Sigma-Aldrich, CA, USA), 1 μM dexamethasone (cat. no. D8893, Sigma-Aldrich, CA, USA), 1 mM IBMX (cat. no. PHZ1124, Thermo Fisher Scientific, CA, USA), and 0.2 mM indomethacin (cat. no. 53861, Sigma-Aldrich, CA, USA) for 16 d. The intracellular lipid droplet accumulation and the adipogenic marker gene mRNA expression were examined using the methods described below.

To investigate the effect of the Wnt agonist, BIO (cat. no. B1686, Sigma-Aldrich, CA, USA) was added to the culture medium at a final concentration of 200 nM, 400 nM, or 800 nM. The dose that has been used in this study was relevant to the previous research that evaluated the effect of BIO on osteogenesis of human multipotent stromal cells [38].

### 4.3. Immunofluorescence Staining

Cells were fixed with 4% buffered formalin at room temperature for 10 min. Cell permeabilisation was performed using 0.15% Triton^®^-X100 in PBS. Horse serum (2% *v*/*v*) was used to inhibit non-specific binding. Cells were stained with β-Catenin XP^®^ Rabbit mAb (cat. no. 8480, Cell Signaling, Danvers, MA, USA) at a 1:100 dilution at 4 °C overnight. Cells were incubated with biotinylated anti-rabbit IgG antibodies (cat. no. 2172707, Sigma-Aldrich, CA, USA) at a dilution of 1:2000 for 40 min. The targeted protein expression was visualised by staining with Strep-FITC (Sigma-Aldrich, CA, USA) at a dilution of 1:500. Nuclei were counterstained with 0.1 μg/mL 4′,6-diamidino-2-phenylindole (TOCRIS Bioscience, Minneapolis, MN, USA). Protein expression and localisation were performed using a fluorescent microscope with an ApoTome system (Carl Zeiss, Oberkochen, Germany). 

### 4.4. Colony-Forming Unit Assay

Cells (500 cells/well in a 6-well plate) were treated with 200 nM, 400 nM, or 800 nM BIO for 14 d. Cells were fixed with 4% buffered formalin for 10 min and stained with Coomassie blue (Sigma-Aldrich, CA, USA). Colony formation was investigated using an inverted microscope (Olympus, Nashville, TN, USA). The staining was eluted with a 5% (*v*/*v*) methanol and 7.5% (*v*/*v*) acetic acid solution. The absorbance was read at 667.5 nm.

### 4.5. In Vitro Scratch Assay

The cells were cultured in 35-mm tissue culture dishes until reaching confluence. A 200 µL pipette tip was used to produce a 100–150 µm cell-free scratch. The cells were treated with 200 nM, 400 nM, or 800 nM BIO and the scratch was observed using an inverted microscope (Olympus, TN, USA) at 24 and 48 h. The dishes were marked to register them to view the same location at both time points. The exposure area was measured using ImageJ software. The percentage of cell migration was calculated. 

### 4.6. Apoptosis Assay

The cells (100,000 cells/well in a 12-well plate) were treated with 200 nM, 400 nM, or 800 nM BIO for 3 d. Annexin V FLUOS Staining Kit (Roche, Indianapolis, IN, USA) was employed to detect early and late apoptosis according to the manufacturer’s protocol. The cells were analysed by a FACS^Calibur^ flow cytometer (BD Bioscience, CA, USA). 

### 4.7. Cell Cycle Analysis

The cells (100,000 cells/well in a 12-well plate) were treated with 200 nM, 400 nM, or 800 nM BIO for 3 d. After trypsinisation, the cells were fixed in 70% ethanol at −20 °C for 30 min. To eliminate RNA, two μL of 4 mg/mL RNase A (cat. no. EN0531, Thermo Fisher Scientific, CA, USA) were added. The cells were stained with 40 μg/mL propidium iodide. After staining, the cells were analysed by a FACS^Calibur^ flow cytometer (BD Bioscience, CA, USA).

### 4.8. Alkaline Phosphatase Staining

The cells were fixed with 4% paraformaldehyde solution for 10 min, followed by incubation with BCIP/NBT tablets (Roche, Branchburg, NJ, USA) for 30 min in a dark environment at room temperature. The ALP-stained cells were observed using an inverted microscope (Olympus, TN, USA).

### 4.9. Alizarin Red S Staining

The cells were fixed with cold methanol for 10 min. The mineral deposition was detected using 2% Alizarin Red S (ARS) staining (Sigma-Aldrich, CA, USA) for 3 min at room temperature. The stained deposition was quantified by solubilising with 10% cetylpyridinium chloride monohydrate in 10 mM sodium phosphate. The destaining protocol was performed at room temperature with gentle agitation for 15 min. The absorbance was measured at 570 nm with a microplate reader (Biotek ELX800, NJ, USA).

### 4.10. Oil Red O Staining

The cells were fixed with 10% buffered formalin for 30 min, followed by incubating with 0.2% Oil Red O solution for 15 min. Lipid accumulation was examined using an inverted microscope (Olympus, TN, USA).

### 4.11. Polymerase Chain Reaction

Total cellular ribonucleic acid (RNA) was extracted using TRIzol reagent (RiboEx solution, cat. no. 301-001, GeneAll, Seoul, Korea). Total RNA (1 μg) was converted to cDNA using the ImProm-II Reverse Transcription System (cat. no. A3800, Promega, WI, USA). Real-time polymerase chain reaction (PCR) was performed using CFX connect Real-Time PCR (Bio-Rad, Singapore) with FastStart Essential DNA Green Master (Roche Diagnostic, Mannheim, Germany). Melt curve analysis was performed to determine product specificity. The mRNA levels of the target genes were normalised to the 18S gene, and the relative gene expression was quantified by the comparative Ct method (2^−ΔΔCt^ method) [102]. The oligonucleotide sequences are shown in Supplementary Table S1. 

### 4.12. RNA Sequencing and Bioinformatic Analysis

Cells were treated with 800 nM BIO for 24 h. Total RNA was extracted using the RNeasy kit (Qiagen, MD, USA), and the high-throughput RNA sequencing was performed at the Omics Science and Bioinformatics Center, Faculty of Science, Chulalongkorn University. The RNA quantity and integrity number were determined using a Nanodrop and an Agilent 2100 BioAnalyzer (Agilent Technologies, CA, USA). RNA (1 µg) was employed for mRNA library construction according to the TrueSeq mRNA stranded library preparation kit (Illumina, CA, USA) protocol. The library quality was examined using the Agilent 2100 Bioanalyzer and Qubit 3.0 fluorometer (Thermo Fisher Scientific, CA, USA). RNA sequencing was performed in the NextSeq. 500 (Illumina, CA, USA). Read quality was checked, trimmed, and filtered by the FastQC and FastQ Toolkit (Illumina, CA, USA) [103]. The RNA sequence reads were mapped with the human reference genome (GRCh38) using a HISAT2 transcript aligner [104,105,106]. Differential expression analysis was examined using the edgeR package [107,108]. The sequencing data were submitted to NCBI’s Gene Expression Omnibus (GSE183633).

### 4.13. Statistical Analysis

All experiments were repeated using cells derived from at least four different donors (*n* = 4). Statistical analysis was performed using Prism 8 (GraphPad Software 8.0, CA, USA). The Mann–Whitney U test was used for two independent group comparisons. For three or more group comparisons, statistical differences were assessed using the Kruskal–Wallis test followed by a pairwise comparison. Significance was defined when *p* < 0.05.

## 5. Conclusions

In conclusion, BIO treatment influenced hDPSC colony formation, cell apoptosis, migration, and osteogenic differentiation. Since a previous report showed that BIO augmented tooth repair in mice models, our results would provide evidence in terms of human cell context. Therefore, BIO-modulated Wnt activation would be an alternative method to promote hDPSC odonto/osteogenic differentiation. To promote dentin bridge formation clinically, BIO could be further developed as a small molecule Wnt agonist-based biomaterial combined in pulp-capping material and would be applied in some type of clinical trial, e.g., pulp-exposed dental cavitation. 

## Data Availability

Any data or material supporting this study’s findings can be made available by the corresponding author upon request. The raw sequencing data can be downloaded from NCBI’s Gene Expression Omnibus (GSE183633).

## Supplementary Materials

The following supporting information can be downloaded at: https://www.mdpi.com/article/10.3390/ijms23158676/s1.
