# Supplementary material for: 6-Bromoindirubin-3′-Oxime Regulates Colony Formation, Apoptosis, and Odonto/Osteogenic Differentiation in Human Dental Pulp Stem Cells
Source: Int J Mol Sci. 2022 Aug 4;23(15):8676. doi: 10.3390/ijms23158676 (PMC9368902; doi:10.3390/ijms23158676)
Supplement: Supplementary file 1 [file ijms-23-08676-s001.zip › ijms-1825290-supplementary.pdf]

6-bromoindirubin-3′-oxime regulates colony formation, apoptosis, and odonto/osteogenic differentiation in human dental pulp stem cells

Chatvadee Kornsuthisopon[1], Sunisa Rochanavibhata[2], Nunthawan Nowwarote[3], Kevin A. Tompkins[4], Waleerat Sukarawan[1], Thanaphum Osathanon[1,5]

[1]Dental Stem Cell Biology Research Unit, Faculty of Dentistry, Chulalongkorn University, Bangkok Thailand 10330

[2]Department of Oral and Maxillofacial Surgery, Faculty of Dentistry, Chulalongkorn University, Bangkok Thailand 10330

[3]Universite Paris Cite, Faculty of Dentistry, Department of Oral Biology, Paris, France and Centre de Recherche des Cordeliers, Sorbonne Universite, INSERM UMRS 1138, Molecular Oral Pathophysiology, Paris, France

[4]Office of Research Affairs, Faculty of Dentistry, Chulalongkorn University, Bangkok THAILAND 10330

[5]Department of Anatomy, Faculty of Dentistry, Chulalongkorn University, Bangkok THAILAND 10330

Corresponding author:

Thanaphum Osathanon, DDS, PhD

Dental Stem Cell Biology Research Unit, Faculty of Dentistry,

Chulalongkorn University,

34 Henri-Dunant Rd. Pathumwan, Bangkok, 10330 Thailand

Tel: +66-2-218-8885, Fax: +66-2-218-8870

Email: thanaphum.o@chula.ac.th

Table S1: The oligonucleotide sequences

Genes	Forward sequences	Reverse sequences	GenBank Accession Number	Size (bp)
GAPDH	5’-TCATGGGTGTGAACCATGAGAA-3’	5’-GGCATGGACTGTGGTCATGAG-3’	NM_002046.3	146
ALP	5’-CGAGATACAAGCACTCCCACTTC-3’	5’-CTGTTCAGCTCGTACTGCATGTC-3’	NM_000478.3	120
ANKH	5’-GAGGTGACAGACATCGTGG-3’	5’-CCTTTAAATCAAGGCCTCTTTCATTAC-3’	NM_054027.6	177
AXIN2	5’-ATGATTCCATGTCCATGACG-3’	5’-CTTCACACTGCGATGCATTT-3’	NM_001363813.1	101
COL1A1	5’-GTGCTAAAGGTGCCAATGGT-3’	5’-ACCAGGTTCACCGCTGTTAC-3’	NM_000088.4	128
DMP1	5’- CAGGAGCACAGGAAAAGGAG -3’	5’- CTGGTGGTATCTTGGGCACT -3’	NM_004407.3	213
DSPP	5’-ATATTGAGGGCTGGAATGGGGA-3’	5’-TTTGTGGCTCCAGCATTGTCA-3’	NM_014208.3	136
ENPP	5’-AAATATGCAAGCCCTCTTTGT-3’	5’-TTTAGAAGGTGGTTAAGACTTCCATGA-3’	NM_006208.3	162
LRP8	5’-AAGTGTGTACCTGCCTCGTG-3’	5’-CACTCGTTCAGCCAGGTAGC-3’	NM_033300.4	92
MMP11	5’-GGCCCTAAAGGTATGGAGCG-3’	5’-TCTCGGTGAGTCTTGGGGAA-3’	NM_005940.5	168
OCN	5’-CTTTGTGTCCAAGCAGGAGG-3’	5’-CTGAAAGCCGATGTGGTCAG-3’	NM_199173.6	166
OSX	5’-GCCAGAAGCTGTGAAACCTC-3’	5’-GCTGCAAGCTCTCCATAACC-3’	NM_001300837.2	161
RUNX2	5’-ATGATGACACTGCCACCTCTGA-3’	5’-GGCTGGATAGTGCATTCGTG-3’	NM_001024630.3	167
LPL	5’-GAGATTTCTCTGTATGGCACC-3’	5’-CTGCAAATGAGACACTTTCTC-3’	NM_000237.3	276
PPARγ	5’-CCAGTGGTTGCAGATTACAAGTATG-3’	5’-TTGTAGAGCTGAGTCTTCTCAGAATAATAAG-3’	NM_138712.5	110
